# Evidence for strain-specific virulence of *Trichomonas gallinae* in African columbiformes

**DOI:** 10.1017/S0031182022001652

**Published:** 2023-02

**Authors:** Jenny C. Dunn, Rebecca C. Thomas, Helen Hipperson, Danaë J. Sheehan, Chris Orsman, John Mallord, Simon J. Goodman

**Affiliations:** 1 RSPB Centre for Conservation Science, Royal Society for the Protection of Birds, The Lodge, Potton Road, Sandy, Bedfordshire SG19 2DL, UK; 2 School of Life and Environmental Sciences, University of Lincoln, Joseph Banks Laboratories, Green Lane, Lincoln LN6 7TS, UK; 3 School of Biology, Irene Manton Building, University of Leeds, Leeds LS2 9JT, UK; 4 NERC Biomolecular Analysis Facility, Department of Animal and Plant Sciences, University of Sheffield, Western Bank, Sheffield S10 2TN, UK

**Keywords:** Feather growth, iron hydrogenase, ITS, *Trichomonas gallinae*, turtle dove

## Abstract

Infection by parasites or pathogens can have marked physiological impacts on individuals. In birds, infection may affect moult and feather growth, which is an energetically demanding time in the annual cycle. Previous work has suggested a potential link between clinically visible *Trichomonas gallinae* infection and wing length in turtle doves *Streptopelia turtur* arriving on breeding grounds. First, *T. gallinae* infection was characterized in 149 columbids from 5 species, sampled on turtle dove wintering grounds in Senegal during the moulting period, testing whether infection by *T. gallinae* is linked to moult. *Trichomonas gallinae* prevalence was 100%, so rather than testing for differences between infected and uninfected birds, we tested for differences in moult progression between birds infected by different *T. gallinae* strains. Twelve strains of *T. gallinae* were characterized at the internal transcribed spacer 1 (ITS1)/5.8S/ITS2 region, of which 6 were newly identified within this study. In turtle doves only, evidence for differences in wing length by strain was found, with birds infected by strain Tcl-1 having wings nearly 6 mm longer than those infected with strain GEO. No evidence was found for an effect of strain identity within species on moult progression, but comparisons between infected and uninfected birds should be further investigated in species where prevalence is lower.

## Graphical abstract

**Figure:**
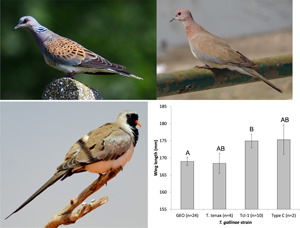

## Introduction

Infection by a parasite or pathogen can have physiological impacts on individuals, even in the absence of clinical signs (Latorre-Margalef *et al*., 2009; Lachish *et al*., 2011; Asghar *et al*., 2015). Infections can initiate trade-offs within individuals because immune defences are a costly investment (Sheldon and Verhulst, 1996); increasing the resources invested in immunity reduces those that can be invested in growth, reproduction and thermoregulation (Lochmiller and Deerenberg, 2000; Sanz *et al*., 2004).

*Trichomonas gallinae* is the causal agent of trichomoniasis, an emerging infectious disease linked to mortality in finches in the UK and Europe (Robinson *et al*., 2010). *Trichomonas gallinae* is historically known as a parasite of columbids and raptors (Stabler, 1954), where it was generally thought to cause few clinical signs (but see Bunbury *et al*., 2008) with the exception of occasional mortality events (Höfle *et al*., 2004; Rogers *et al*., 2018). However, the emergence of a novel strain (termed the type A strain; Gerhold *et al*., 2008) linked to the finch epizootic (Lawson *et al*., 2011) has been associated with mortality in adult and nestling European turtle doves *Streptopelia turtur* (Stockdale *et al*., 2015), in which *T. gallinae* is found at very high prevalence in the UK (86%: Lennon *et al*., 2013; 100%: Stockdale *et al*., 2015) and Europe (93% from samples collected using standard *T. gallinae* sampling and culture techniques: Marx *et al*., 2017).

Moult is an energetically demanding stage in a bird's annual cycle (Rubolini *et al*., 2002), with trade-offs demonstrated within individuals overlapping moult and other energetically demanding activities such as breeding or migrating, compared to individuals undergoing these processes sequentially (Rubolini *et al*., 2002; Echeverry-Galvis and Hau, 2013). Trade-offs between moult and immunity have also been identified (Moreno-Rueda, 2010). For example, Laysan albatrosses *Phoebastria immutabilis* with a higher nematode burden began primary moult later, replaced fewer primary feathers and grew fewer feathers at a time (Langston and Hillgarth, 1995). Malarial coinfections reduce feather growth in house martins *Delichon urbica* (Marzal *et al*., 2013), although no effect of single haemosporidian infections was found on the progression of moult in captive yellowhammers *Emberiza citrinella* (Allander and Sundberg, 1997). However, trade-offs may be context-dependent: for example; *Leucocytozoon ziemanni* infection is associated with reduced clutch size in Tengmalm's owls *Aegolius funereus* only in low food abundance years (Korpimäki *et al*., 1993).

Previous work found that turtle doves arriving on UK breeding grounds with clinical signs (infected by the type A strain responsible for the finch epizootic) of trichomonosis had markedly shorter wings than those without clinical signs (but still carrying the parasite; Stockdale *et al*., 2015), suggesting a potential relationship between infection with the lethal type A strain and restricted moult. Here, we first investigated the prevalence and strain identity of *T. gallinae* in columbids on turtle dove wintering grounds in Senegal, screening migratory turtle doves alongside resident laughing doves *Spilopelia senegalensis*, black-billed wood doves *Turtur abyssinicus*, Namaqua doves *Oena capensis* and vinaceous doves *Streptopelia vinacea*. Second, we tested whether infection by different strains of *T. gallinae* influences either wing length or moult within species, to test whether sub-clinical *T. gallinae* infection might restrict moult in columbids.

## Methods

### Study sites and field data collection

Columbids were caught at a turtle dove wintering roost near Sandiara, Senegal (14°24′N, 16°47′W) during February–March 2014, and January–March 2015. Birds were caught using mist nets at roost sites and near watering holes within an area of regenerated acacia scrub fenced off to protect against livestock grazing. Once caught, birds were weighed using a digital balance (±0.1 g), their maximum wing length measured (Redfern and Clark, 2001; ±0.5 mm) and they had an oral swab taken to test for the presence of *T. gallinae* prior to release. A swab (4 mm diameter for turtle doves, vinaceous doves and laughing doves; 2.5 mm diameter for black-billed wood doves and Namaqua doves) was moistened using sterile water, and passed gently down the oesophagus and into the crop, where it was passed through 2 figure of 8 motions before being gently removed and inoculated into an In Pouch™ culture kit (Biomed Diagnostics, Oregon, USA). Culture kits were incubated at 37°C for 3–7 days (Bunbury *et al*., 2005) and then processed as detailed below. All birds appeared healthy when caught, with no visible signs of trichomonosis (caseous lesions visible in the oral cavity, matted feathers or saliva around the bill, apparent difficulties swallowing, thin with protruding breastbone) or any other clinical signs of disease.

### Parasite isolation

Following incubation, *T. gallinae* parasites were either isolated (2014 samples) or mixed 1:1 with 100% ethanol and shipped to the UK prior to parasite isolation (2015 samples). Parasites were isolated following the protocol of Riley *et al*. (1992), modified as follows: 2.5 mL of culture or culture/ethanol mix was centrifuged at 3200 rpm for 5 min, then the resulting pellet was washed with 1 mL of phosphate-buffered saline (PBS) by centrifugation and re-suspended in 200 *μ*L PBS. Samples were stored at −20°C until DNA extraction.

### DNA extraction and detection of parasites

DNA extraction was carried out using a modified ammonium acetate protocol (Nicholls *et al*., 2000). Briefly, the parasite pellet was digested overnight in digestion buffer (20 mm EDTA, 50 mm Tris, 120 mm NaCl, 1% SDS, pH 8.0) with 50 *μ*g of proteinase K. Ammonium acetate (4 m) was then used to precipitate out the proteins, and ethanol precipitated out the DNA. The resulting DNA pellet was dissolved in 20–50 *μ*L low TE buffer, depending on the size of the pellet, in a water bath at 65°C. The extracted DNA was stored at −20°C. Samples were not all individually quantified but DNA concentrations based on a subset of samples typically ranged from 0.5 to 60 ng *μ*L^−1^.

Two polymerase chain reactions (PCRs) were carried out for each sample, 1 targeting a 400 bp length of the internal transcribed spacer (ITS) ribosomal region using the primer pair TFR1 (5′-TGCTTCAGTTCAGCGGGTCTTCC-3′) and TFR2 (5′-CGGTAGGTGAACCTGCCGTTGG-3′) (Gaspar da Silva *et al*., 2007), and the second targeting a 1000 bp fragment of the iron hydrogenase (*Fe-hyd*) gene using the primer pair TrichhydFOR (5′-GTTTGGGATGGCCTCAGAAT-3′) and TrichhydREV (5′-AGCCGAAGATGTTGTCGAAT-3′) (Lawson *et al*., 2011) to allow for the identification of sub-types. All PCRs were run on either a GeneAmp 9700 PCR system (Applied Biosystems, Foster City, CA, USA) or a DNA Engine Tetrad 2 (Bio-Rad Laboratories Inc., Hercules, CA, USA), and a negative control of molecular grade water and a positive control were included in each PCR run. ITS PCRs were carried out in a 10 *μ*L reaction volume comprising 0.8× Qiagen multiplex PCR MasterMix (Qiagen, Hilden, Germany), 0.5 *μ*m each of forward and reverse primers and 1 *μ*L template DNA. The touchdown PCR protocol consisted of an initial 15 min denaturation at 95°C, followed by 11 cycles of 60 s at 94°C, 30 s at 66°C decreasing by 1°C per cycle and 60 s at 72°C, then 24 cycles as before but at an annealing temperature of 55°C, with a final 10 min extension step at 72°C. The *Fe-hyd* PCR consisted of: 1× PCR buffer (Promega, Southampton, UK), 3 mm MgCl_2_ (Promega, Southampton, UK), 0.25 *μ*m dNTP mix (Promega, Southampton, UK), 0.25 *μ*m forward and reverse primers (Invitrogen, Hertford, UK), 5 U *μ*m^−1^ Go Taq Hot Start polymerase (Promega, Southampton, UK) and a volume of molecular grade water to make the total PCR volume to 49 *μ*L whereby 1 *μ*L of DNA was then added. PCR thermal cycling was performed as follows: 5 min denaturation at 94°C, then 35 cycles of 45 s at 94°C, 30 s at 53°C and 45 s at 72°C, followed by 5 min at 72°C for a final elongation. All PCR products were electrophoresed through a 1.5% agarose gel, which was stained with Gel Red, in 1× TBE buffer and visualized under UV light.

Positive PCR products were either purified using Wizard SV Gel & PCR Clean-Up System (Promega, Southampton, UK) and sent for bidirectional Sanger sequencing by Beckman Coulter Genomics (Takeley, Essex, UK) (*n* = 52), or prepared for sequencing on an Illumina MiSeq (*n* = 101) as part of a wider project (Thomas, 2017; Thomas *et al*., 2022). Details of preparation for Illumina sequencing are published in detail elsewhere (Thomas *et al*., 2022), and summarized in the Supplementary material.

### Sequence analysis

Sequences returned from Sanger sequencing were manually assessed for sequencing errors, trimmed and aligned in BioEdit (Hall, 2005). Analysis of MiSeq sequences is described in detail elsewhere (Thomas *et al*., 2022), and available in the Supplementary material. Following processing, all sequences were queried using the NCBI-BLAST algorithm (Altschul *et al*., 1997) to assign strain identity.

### Moult score

Each of the 10 primary feathers on the right wing of each bird was examined to determine the stage of moult, and each bird's moult was scored between 0 (moult not started; all feathers old) and 50 (moult completed; all feathers new). Each feather was scored between 0 (old feather present) and 5 (new feather completely grown) depending on the proportion of total primary feather length emerged from the feather sheath, and the totals summed to provide an index of the stage of moult.

### Statistical analysis

To determine whether *T. gallinae* strain influenced wing length or moult, we analysed data for each species separately where sample sizes and strain variation were sufficient. Namaqua doves were excluded from analysis because all but 3 individuals carried the same parasite strain, and vinaceous doves were not analysed because we only identified the strain from 1 individual. For the remaining 3 species separately, we removed any strains represented in only 1 bird, and constructed linear models with wing length or moult score as the response variable and strain type and day (to control for the progress of moult throughout the winter) as predictor variables. We then tested the significance of each variable by removing each variable in turn from the full model and comparing models with and without the variable. Residuals were checked for homoscedasticity throughout and response variables transformed where appropriate.

## Results

A total of 149 columbids were caught over 2 winters: 55 European turtle doves, 43 Namaqua doves, 34 laughing doves, 15 black-billed wood doves and 2 vinaceous doves.

### *Trichomonas gallinae* prevalence and identity

**Fig. 1.**
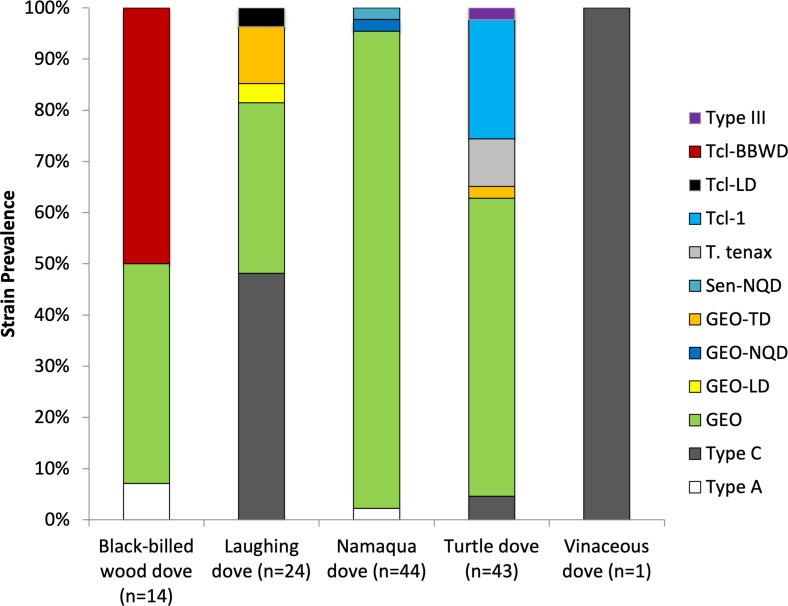
*Trichomonas gallinae* strain prevalence in African columbid species, for individuals from which good quality sequence was obtained.

All birds tested were positive for the presence of *T. gallinae*. We obtained good quality sequence from 119 birds (81%; 13 black-billed wood doves; 23 laughing doves; 41 Namaqua doves; 41 turtle doves and 1 vinaceous dove; Fig. 1); prevalence and sequence data for turtle doves has been reported elsewhere (Thomas *et al*., 2022), but are summarized below for comparison. The most common strain present, GEO, was identical to one previously isolated from Australasian columbids [GenBank accession number (hereafter A/N) JQ755287], and was found in 6 black-billed wood doves, 9 laughing doves, 41 Namaqua doves and 25 turtle doves. A new strain, with 99% match to the GEO strain and named GEO-TD was found in 1 turtle dove and 3 laughing doves (A/N OM417014), 2 of which were coinfected with type C (see below). Turtle doves had 3 strains only found in this species, 4 carrying a strain matching *Trichomonas tenax* (A/N U86615), 10 carrying a strain matching Tcl-1 (A/N KF993705) and 1 carrying a strain matching type III (A/N FN433473). Seven black-billed wood doves carried a new strain not found in other species with 99% match to Tcl-1, and named Tcl-BBWD (A/N OM417010). The type C strain (A/N EU215362) was found in 13 laughing doves (including 2 coinfected with GEO-TD, see above), 2 turtle doves and 1 vinaceous dove, and the type A strain (A/N GQ150752) was found in 1 black-billed wood dove and 1 Namaqua dove. Two Namaqua doves were each infected with new strains GEO-NQD (A/N OM417020) and Sen-NQD (A/N OM417018), and 1 laughing dove was coinfected by 2 new strains: GEO-LD (A/N OM417015) and Tcl-LD (A/N OM417016).

Further identification of sub-types classified by the *Fe-hyd* gene was possible for 13 birds. We identified 1 known type A, sub-type A2, in a black-billed wood dove (A/N ON936876); this strain had previously been isolated from a Madagascar turtle dove *Streptopelia picturata* (A/N JF681141). We identified 5 C sub-types, of which 3 are reported for the first time here. We isolated the C6 sub-type from 2 laughing doves (A/N ON936878): this sub-type has previously been reported from a booted eagle *Hieraaetus pennatus* in Spain (A/N KP099941). As previously reported (Thomas *et al*., 2022), we identified sub-type C8-TD (A/N MT418242) from 1 turtle dove. All 3 novel sub-types were identified from laughing doves, with C9-LD (A/N ON93689), C10-LD (A/N ON936881) and C12-LD (A/N ON936875) isolated from 5, 1 and 1 individual, respectively; C9-LD (A/N ON936880) was also isolated from 1 vinaceous dove. We identified 2 Tcl-1 sub-types (T1-TD, A/N MT418249 and T2-TD, A/N MT418246), both reported in turtle doves in Senegal as part of a separate paper from this study (Thomas *et al*., 2022). Finally, we identified a sub-type for the new ITS strain Tcl-BBWD, which we designated NT1-BBWD (A/N ON936877), isolated from 2 black-billed wood doves.

### Associations with wing length and moult

**Fig. 2.**
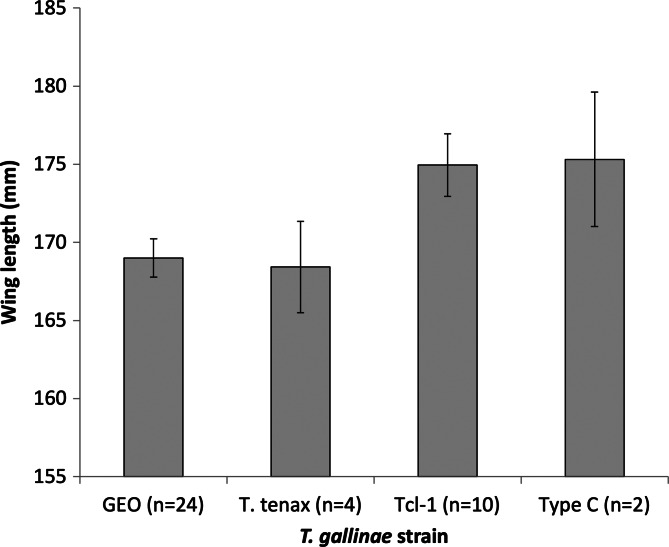
*T. gallinae* strain was associated with wing length in turtle doves with marginal significance (Table 1). Differing letters above bars denote marginally significant differences (0.05 < *P* < 0.10). Bars show predicted mean ± s.e. from the linear model, with median value of day (30th January).

All birds were infected with *T. gallinae*, so we could not test for an effect of infection *per se* on wing length or moult. No associations were found between *T. gallinae* strain and moult score for any of the 3 species examined (Table 1a). Turtle doves infected by the GEO strain had wings 5.94 mm shorter on average (with marginal significance: *P* = 0.052; overall wing length range 159–180 mm) than those infected by the Tcl-1 strain (Fig. 2) when controlling for an increase in moult score with day (Table 1b). No significant associations were found for laughing doves or black-billed wood doves (Table 1b).

**Table 1.** Results of linear models predicting (a) moult score and (b) wing length in 3 dove species according to *Trichomonas gallinae* strain, controlling for day

	Turtle dove			Laughing dove			Black-billed wood dove		
	Sum of square	*F*	*P*	Sum of square	*F*	*P*	Sum of square	*F*	*P*
(a) Moult score									
*T. gallinae* strain	42.99	0.18	0.91	89.47	0.76	0.51	60.81	0.80	0.47
Day	**713**.**43**	**8**.**90**	**0**.**006**	68.45	1.17	0.33	136.90	1.80	0.31
(b) Wing length									
*T. gallinae* strain	*288*.*05*	*2*.*83*	*0*.*052*	47.66	0.92	0.41	3.75	0.55	0.47
Day	**203**.**69**	**6**.**01**	**0**.**019**	57.90	2.25	0.15	0.07	0.01	0.92

Statistics relate to the comparison of models and without each term, presenting the sum of squares, *F* and *P* values. Terms highlighted in bold are significant at *p* < 0.05; those in italics are marginally significant at 0.05 < *p* < 0.1.

## Discussion

We confirm a high prevalence of infection by *Trichomonas* spp. in West African columbids, from both resident and migrant species, with multiple parasite strains circulating in host populations. To our knowledge, this is the first investigation of sub-clinical *Trichomonas* infection in West African birds, and adds to the growing literature on this parasite, which may be a cause of conservation concern in some host species (Bunbury *et al*., 2007; Stockdale *et al*., 2015). We find some evidence of association between *Trichomonas* strain and wing length in turtle doves, suggesting further research to test for differential effects of *Trichomonas* strain type on host physiology is warranted.

We found 12 *Trichomonas* strains circulating in West African columbids, with further genetic variation at the *Fe-hyd* region identified within 2 of these strains (type C and Tcl-1). This level of strain diversity in a single location is notable, and higher than that seen in columbids in the UK, either from dead or shot birds (4 strains at the ITS region; Chi *et al*., 2013) or from live-sampled columbids (4 strains at the ITS region; Lennon *et al*., 2013). Turtle doves in our study had the highest strain diversity with 6 strains. As long-distance migrants, they may be exposed to infection over a wider geographical area and thus be exposed to a higher diversity of parasite strains (Koprivnikar and Leung, 2015). Only 1 additional strain, type A, was found in a larger-scale study of 131 turtle doves covering Senegal, Burkina Faso, France and the UK (Thomas *et al*., 2022). Laughing doves also showed high strain diversity with 5 strains isolated from only 24 birds (Fig. 1).

The most common strain we found, accounting for 63% of infections and found in all species apart from the vinaceous dove, was the GEO strain, previously identified from multiple dove and pigeon species across Europe and into the Middle East (Marx *et al*., 2017; Rajabloo, M. *et al*., unpublished). The next most common strain was type C, accounting for 12% of infections, previously widely reported from doves and pigeons in Europe, the Middle East, China and the USA (e.g. Gerhold *et al*., 2008; Marx *et al*., 2017; Feng *et al*., 2018; Arabkhazaeli *et al*., 2020). Tcl-1, a strain similar to *Trichomonas canistome*, accounted for 8% of infections overall but was found in turtle doves only in this study. Previous studies have identified this strain only in turtle doves in Europe and Africa, and in stock doves *Columba oenas* in Europe (Martínez-Herrero *et al*., 2014; Marx *et al*., 2017; Thomas *et al*., 2022). The only other strain found in more than 5% of individuals was a novel strain with the highest similarity to Tcl-1 (*T. canistome*-like), which we designated Tcl-BBWD. This strain was only found in black-billed wood doves in this study. Given that the majority of Tcl-1 occurrences have been found in a single host species, it may be that Tcl-like strains have a higher degree of host specificity than other strains of *T. gallinae*.

We found a low rate of coinfection by multiple parasite strains, finding 3 of 34 laughing doves to be infected by 2 strains each; coinfection was not detected in the other species examined. This is a similar finding to that in a larger-scale study of turtle doves only, where only 1% of individuals were found to be coinfected by multiple strains of *T. gallinae* (Thomas *et al*., 2022), and may suggest either within-host competition between *T. gallinae* strains, or high mortality of coinfected individuals (see Thomas *et al*., 2022 for a further discussion of potential reasons behind this finding).

Interestingly, we did not find type A strain in the migratory turtle dove, but did isolate this strain from resident black-billed wood and Namaqua doves. Sequencing at the *Fe-hyd* region indicated that – at least in the black-billed wood dove – this is not the same strain as is responsible for the finch epizootic (Robinson *et al*., 2010; Lawson *et al*., 2011). To our knowledge, this is the first report of a type A strain from the African continent, although a strain identical at the ITS region to the one we found has been reported from columbids in Mauritius (Gaspar da Silva *et al*., 2007). Current knowledge suggests that type A *T. gallinae* strains are more likely to be associated with clinical signs and mortality than other strains (Sansano-Maestre *et al*., 2009; Lawson *et al*., 2011; Lennon *et al*., 2013; Stockdale *et al*., 2015), and a type A strain has been associated with morbidity and mortality in turtle doves in the UK (Stockdale *et al*., 2015). Given the low prevalence of type A found in our study, we could not test whether this strain was associated with reduced wing length as suggested by Stockdale *et al*. (2015).

We found turtle doves infected by the GEO strain to have wings nearly 6 mm shorter on average than those infected by Tcl-1, but found no evidence for associations between wing length and strain in laughing doves or black-billed wood doves. Our sample size for other strains was much smaller, so our study may not have had sufficient statistical power to detect any effects of infection by other strains. As long-distance migrants, it may be that turtle doves have a relatively short time in which to moult, and thus may be more susceptible to any physiological impacts of infection from more virulent parasite strains upon wing length than resident species. However, in passerines, long-distance migrants spend a similar length of time completing moult compared to residents (Kiat *et al*., 2019). Whilst these data are not readily available for columbids, 60–80% of turtle doves previously caught on migration had begun primary moult on breeding grounds and suspended moult during migration to complete on wintering grounds (Mead and Watmough, 1976; Swann and Baillie, 1979) suggesting that primary moult may occur over a relatively prolonged period.

Our data suggest that Tcl-1 might be a specialist strain, with GEO being more generalist and found within all host species for which we sequenced *Trichomonas* from more than 1 individual. This strain has also been isolated from passerines and seed food resources in the UK (Thomas *et al*., 2022). Our data are consistent with the suggestion that generalist parasites may be more virulent, or have a greater impact on their hosts, than specialist parasites (Leggett *et al*., 2013), with the seemingly more generalist GEO exhibiting a marginally higher impact on the host (shorter wing length) than the seemingly more specialist Tcl-1. Whilst a larger sample size of birds infected by these and other strains would be necessary to draw any firm conclusions, our data suggest that further investigation may be warranted. Data from populations with parasite prevalence <100% would greatly improve our overall understanding of any impacts of parasite infection *per se*. Similarly, studies carried out in populations where individuals could be caught and sampled regularly over an extended time period would be extremely valuable in elucidating the epidemiology and potential within-host strain turnover of *T. gallinae* parasites.

## Data Availability

Sequence data are available from GenBank under accession numbers OM417009-021 (ITS) and ON936875-881 (*Fe-hyd*), and through the Sequence Read Archive under accession numbers SAMN31742540-617 (BioProject PRJNA578480). Raw data and analysis code will be made available through an open access repository after acceptance.

## Supplementary material

For supplementary material accompanying this paper visit https://doi.org/10.1017/S0031182022001652. click here to view supplementary material
