# Supplementary material for: Evidence for strain-specific virulence of Trichomonas gallinae in African columbiformes
Source: Parasitology. 2022 Dec 19;150(2):206–11. doi: 10.1017/S0031182022001652 (PMC10090629; doi:10.1017/S0031182022001652)
Supplement: Supplementary file 1 [file S0031182022001652sup001.docx]

Evidence for strain-specific virulence of *Trichomonas gallinae* in African Columbiformes – Supplementary Material

Jenny C. Dunn, Rebecca C. Thomas, Helen Hipperson, Danaë J. Sheehan, Chris Orsman, John Mallord, Simon J. Goodman

**Preparation of samples for Illumina Sequencing**

First, Illumina sequencing primer sites (F: 5’-TCTACACGTTCAGAGTTCTACAGTCCGACGATC-3’ and R: 5’–GTGACTGGAGTTCAGACGTGTGCTCTTCCGATCT-3’) were added in a 25 μl PCR mix. This comprised 10 µl QIAGEN Multiplex PCR Master Mix, 2.5 µl of each forward and reverse primer (3 µM) tailed with Illumina sequencing primer sites (F: 5’-TCTACACGTTCAGAGTTCTACAGTCCGACGATC-3’ and R: 5’–GTGACTGGAGTTCAGACGTGTGCTCTTCCGATCT-3’) and 1 µl of DNA. The PCR thermal cycling programs were as above. Amplicons for each sample were normalised according to the intensity of the PCR product on a 1% agarose gel stained with ethidium bromide. The second PCR added sample-specific indexes: the 10 µl reaction volume consisted of 5 µl QIAGEN Multiplex PCR Master Mix, 1 µl of each Illumina Fi5 and Ri7 indexes (1 µM) whose combination was specific to each well (Integrated DNA Technologies, Belgium) and 4 µl of the PCR product from PCR1. The thermal cycler conditions were as follows: 15 min at 95°C, then 10 cycles of 10 s at 98°C, 30 s at 65°C and 30 s at 72°C, finishing with 5 min at 72°C. Samples were normalised after quantifying on a FLUOstar OPTIMA (BMG Labtech, Ortenberg, Germany) using the QuantiFluor® dsDNA system (Promega, UK) following the manufacturer’s instructions. The Agencourt AMPure XP system (Beckman Coulter, UK) was used for purification according to the manufacturer’s instructions. Purified products were eluted in 15 µl of nuclease- free TE (10 mM Tris-HCl, 1 mM EDTA, pH 8.0) with 1.5 µl of 10 mM Tris-HCl/ 0.05% Tween 20 (pH 8.0) added. The prepared libraries were checked on the Agilent 4200 Tapestation (Agilent Technologies, CA, USA) for the expected peak amplicon size.

Quantification of each pooled product was performed using qPCR. Triplicate dilutions of 1:100, 1:1000 and 1:10000 of the libraries were produced by serial dilution. The reagent mix and thermal cycling conditions were performed as per the manufacturer’s instructions (KAPA library quantification kit, KAPA Biosystems, UK). A StepOnePlus Real- Time PCR system (Applied Biosystems, CA, USA) was used to run the qPCR. The concentration of each library was calculated using the KAPA data analysis template and normalised to 4 nM. The library was sequenced using 250 paired-end reads on a MiSeq desktop sequencer (Illumina, San Diego, CA), over four different Miseq runs, in combination with other samples as part of a larger project (Thomas, 2017). Each run contained at least 10% duplicates (i.e. 10 duplicates per 96 well plate).

**Illumina sequence analysis**

MiSeq sequences were demultiplexed into sample files according to Fi5 and Ri7 indexes by the Illumina MiSeq control software (V 2.5.0.5). Trimmomatic v0.36 (Bolger *et al.*, 2014) was used to remove Illumina adapter sequences, low quality bases in the leading or trailing ends and low quality sequences that did not meet the minimum Phred quality score of 20 or the minimum length of 100 bp. Paired end reads were aligned using FLASH 1.2.11 (Magoč and Salzberg, 2011); sequences that did not meet the minimum length of 250 bp were discarded. Sequences from multiple amplicons (ITS and *Fe-hyd*) were demultiplexed according to the primer sequences using jMHC and the output file gave sequence variant depths quantified among amplicons (Stuglik *et al.*, 2011). An approach known as the DOC (Degree Of Change) was used to distinguish between biologically accurate sequences and artefacts, based on the frequency of sequence variants found per sample (Lighten *et al.*, 2014). A variant had to be present in at least 50 copies within a sample to be retained within the analysis. This value was chosen to minimize the risk of false positives.
